# Human Chronotypes from a Theoretical Perspective

**DOI:** 10.1371/journal.pone.0059464

**Published:** 2013-03-27

**Authors:** Adrián E. Granada, Grigory Bordyugov, Achim Kramer, Hanspeter Herzel

**Affiliations:** 1 Institute for Theoretical Biology, Humboldt University, Berlin, Germany; 2 Laboratory of Chronobiology, Charité Universitätsmedizin, Berlin, Germany; Vanderbilt University, United States of America

## Abstract

The endogenous circadian timing system has evolved to synchronize an organism to periodically recurring environmental conditions. Those external time cues are called Zeitgebers. When entrained by a Zeitgeber, the intrinsic oscillator adopts a fixed phase relation 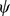 to the Zeitgeber. Here, we systematically study how the phase of entrainment depends on clock and Zeitgeber properties. We combine numerical simulations of amplitude-phase models with predictions from analytically tractable models. In this way we derive relations between the phase of entrainment 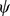 to the mismatch between the endogenous and Zeitgeber period, the Zeitgeber strength, and the range of entrainment. A core result is the “180° rule” asserting that the phase 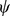 varies over a range of about 180° within the entrainment range. The 180° rule implies that clocks with a narrow entrainment range (“strong oscillators”) exhibit quite flexible entrainment phases. We argue that this high sensitivity of the entrainment phase contributes to the wide range of human chronotypes.

## Introduction

The circadian clock possesses two core properties. The first one is the endogeneity of circadian rhythms, and consequently the existence of a well-defined natural period 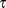, which expresses itself under constant environmental conditions. The second property is the capability to synchronize to a periodic external Zeitgeber by establishing a precise phase relation to it. This paper applies mathematical modeling to connect these two properties and to explain in a systematic way how the timing between the clock and its Zeitgeber is determined by clock and Zeitgeber parameters.

Without external time cues, which are called Zeitgebers, a clock runs with its characteristic period 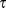, which may deviate from 24 h. On the other hand, many Zeitgebers on the Earth are quite precise and possess a “dian” period 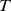 of exactly one day. If the strength of the Zeitgeber is capable to overcome the period mismatch 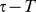, the Zeitgeber enforces the periodicity of 24 h in the clock. This situation is called synchronization or entrainment. The range of the period mismatches 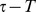 for which synchronization occurs is called entrainment range. In fact, it is the *difference* of both periods 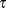 and 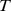 (but not the single periods *per se*) that determines whether the clock would be synchronized or not for a given Zeitgeber strength.

In a synchronized situation, the intrinsic clock adopts a stable phase relation to the Zeitgeber. The difference 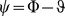 between the phase of the Zeitgeber 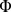 and the phase of the clock 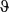 attains a well-defined value called “phase of entrainment”. A large body of research has been accumulated on the phase of entrainment, since it is critical for the coordination of daily rhythms in physiology, metabolism, and behavior [1]. Variations of the phase of entrainment has been extensively studied in the past decades [2], [3], [4], [5]. From an evolutionary perspective, the phase of entrainment is a parameter under selection and, hence, a tight regulation is expected [6].

The goal of this paper is to study systematically how the phase of entrainment depends on the clock and Zeitgeber properties. We approach this problem by theoretical studies of analytically tractable models, supported by numerical simulations. Our main result relates the phase of entrainment 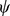 to the period mismatch 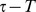, Zeitgeber strength, and the range of entrainment. A cornerstone of this theory is the “180° rule” asserting that within the range of entrainment, the entrainment phase can vary over a range of about 180°. A similar behaviour of entrainment phase has been already noticed in earlier studies [1], [7], [8]. Here we explain how the 180° rule can be rigorously derived from three quite different modeling approaches. We additionally discuss the applicability of the rule and point out its limitations.

The 180° rule implies that clocks with a narrow range of entrainment exhibit high sensitivity of entrainment phase to the mismatch between endogenous and Zeitgeber periods. We argue that this high sensitivity of entrainment phase contributes to the wide range of chronotypes found in humans [4], [9]. Furthermore, our conceptual framework provides insight how midnight-locked, dusk-locked, and dawn-locked entrainment phases can be obtained [2], [5]. Our theory predicts also the dependence of entrainment phase on Zeitgeber strength (see [10] for experimental data).

On the theoretical side, our results bridge discrete and continuous approaches to circadian entrainment. Both time-discrete, PRC/PTC-based models and time-continuous models for the phase of clock lead essentially to the same dependencies of entrainment phase on oscillator and Zeitgeber parameters.

### Mathematical Model

A hierarchy of mathematical models has been developed to describe circadian oscillators ranging from van der Pol oscillators [7] to comprehensive biochemical models [11], [12]. Basic features of entrainment, however, can be studied with the help of simple yet generic amplitude-phase models [13]. We assume that the oscillator of interest is characterized by its amplitude, its intrinsic period, and its stability with respect to amplitude perturbations. These general oscillator properties can be parameterized by the Poincaré oscillator [14], [15], which is given in polar coordinates with radius 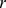 and phase 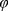 by
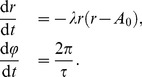
(1)


Here, the parameters 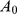 and 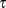 denote amplitude and intrinsic period of the oscillator. The ratio 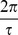 is the angular frequency of the oscillator. The parameter 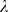 quantifies the relaxation of perturbations to the stable limit cycle with 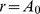. Small values of 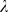 correspond to slow and large values of 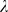 to fast amplitude relaxation to the stationary value 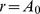. Two characteristic values of 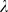 were used: 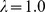 for “strong” oscillators and 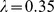 for “weak” oscillators, see below.

**Figure 1:**
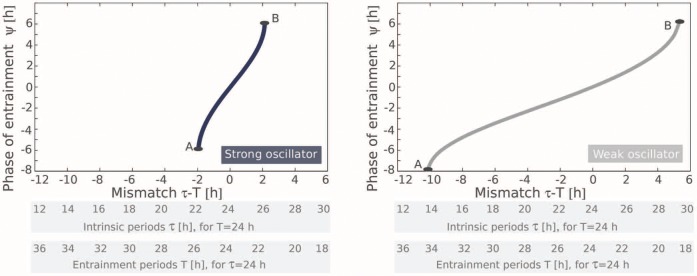
Dependences of entrainment phase 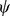 on period mismatch 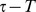 between endogenous and Zeitgeber periods. Left: strong oscillator with narrow entrainment range resulting in high sensitivity of the entrainment phase on mismatch. Right: weak oscillator with wide entrainment range and smaller slope of the function 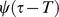.

We have shown recently that oscillators with large amplitude 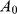 and large relaxation rate 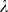 exhibit narrower entrainment ranges [10]. Such oscillators will be termed “strong” in this paper (compare Figure 1). We will show below that strong oscillators are characterized by quite flexible entrainment phase.

## Results

### Entrainment Phase for Strong and Weak Oscillators

The framework that we are going to present can be applied to the circadian clock on the organismic level [8] as well as in different tissues [16], [17]. In both cases, a large range of entrainment phases has been reported. Recently, temperature cycles were described as a universal entrainment clue for different tissues [18]. It has been shown that the mammalian pacemaker, the suprachiasmatic nucleus (the SCN), is quite resistant to phase resetting. Contrarily, pituitary and lung cultures were found to be easily entrainable [10], [18].

The low and high susceptibility of circadian oscillators to Zeitgebers leads to the concept of strong and weak oscillators [8], [10]. Weak oscillators have a wide entrainment range, tolerating large mismatches to Zeitgeber periods. Lung tissues, as an example of a weak oscillator, can be entrained by alterations between 37°C and 35°C using Zeitgeber periods of 20 and 28 h [10]. In contrast, the suprachiasmatical nucleus is quite entrainment-resistant [10], [18] and has, consequently, a narrow range of entrainment. The distinction of strong and weak oscillators can be also applied to clocks on the organismic level. For example, Aschoff and Pohl in [8] suggest that vertebrates exhibit narrow entrainment ranges compared to unicellular organisms and plants. These differences might be due to differential responsiveness to the entrained signal [8] and/or due to different feedback couplings [8], [10]. Mathematically, strong oscillators are characterized by large oscillation amplitudes, fast relaxation of perturbations, and small-amplitude PRCs [10].

As discussed above, the value of the period mismatch 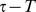 determines whether the clock would be entrained or not. Thus we can on equal footing study mismatches due to varying endogenous period 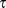 (different phenotypes) as well as experimental variations of Zeitgeber periods 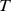 [6]. Positive mismatches 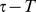 result, e.g., from driving the circadian oscillator with a short Zeitgeber period 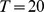 h. Negative mismatches correspond to 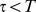. For example, a patient with the Familial Advanced Sleep Phase Syndrome (FASPS) had a short endogenous period of 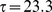 h [19] leading to a mismatch of 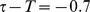 h.

Figure 1 shows the phase of entrainment 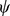 as a function of the mismatch 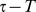 for strong and weak oscillators. The points 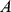 and 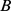 mark the borders of the entrainment range. Our simulations reproduce the observations that the entrainment phase increases with mismatch 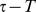 [1]. This implies, for example, that organisms with a short endogenous period have earlier activity phases. For a given endogenous period 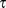, we find early peaks for Zeitgeber period 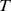 longer than 24 h [6].

Both strong oscillators (left) and weak oscillators (right) reproduce the expected increase of 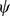 with mismatch 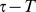. An obvious difference is the steepness of the increasing function 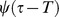. The strong oscillator (left) exhibits a high sensitivity of the entrainment phase 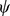 to changes of the mismatch. In the following, we relate this finding to properties of the oscillator and its Zeitgeber. The slope of the curves in Figure 1 is determined by the width of the entrainment range and the vertical phase span. The vertical variation is in both cases about 12 h. The entrainment range, however, is quite different. Consequently, the slope of the curve is nearly reciprocal to the width of the entrainment range. In other words, strong oscillators with a narrow entrainment range (see Figure 1, left panel) exhibit a quite flexible entrainment phase.

This strong dependence of the phase sensitivity on the entrainment range is based on the observation that both oscillator types exhibit a 12 h range of entrainment phases. A 12 hours variation corresponds to 180° for periods of about 24 hours. Our simulations in Figure 1 suggest that a 180° variation of phase 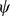 within the entrainment range is a common feature of periodically driven oscillators. Empirical data from many organisms [8] support the theoretical prediction [7] that the entrainment phase spans a range of about 180°. We derive below this “180° rule” using discrete and continuous models of entrainment.

### Is there a 180° Rule?

The most striking result of the previous section is that for both strong and weak oscillators, the phase can vary over the same range of about 180° across the entrainment range. Here, we provide an explanation for this fact based on three different approaches: phase response curves, the Kuramoto equation for the phase of entrainment and a forced linear damped oscillator. All three approaches result in similar phase flexibility under variation of the period mismatch. Note that these considerations are applied to oscillators with relatively narrow entrainment ranges, characteristic for many vertebrates including humans [20], [21].

#### Phase response curves

Phase response curves (PRCs) are an established theoretical tool in chronobiology [20]. For a circadian oscillator, a PRC describes the amount of phase shift, caused by a short Zeitgeber pulse in dependence on when the Zeitgeber pulse is applied.

**Figure 2:**
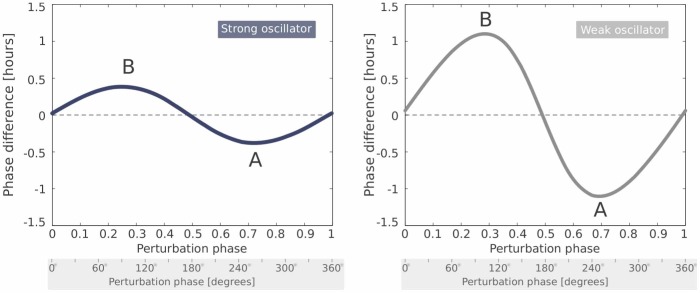
Phase response curves of strong (left) and weak (right) oscillators. The extrema marked by A and B are related to the borders of the entrainment range (compare Figure 1) as explained in the text.

In Figure 2 we plot numerically obtained PRCs of the strong and weak oscillators from Figure 1. In both cases, there are positive and negative phase shifts: During the first half of the circadian cycle, a short Zeitgeber pulse instantaneously advances the clock, whereas in the second half a Zeitgeber pulse delays the clock. The strong oscillator (on the left) has a PRC with a smaller amplitude compared to the weak one. Being phase variables, phase shift and the relative position of the pulse can be specified either in hours or in degrees or radians.

In order to entrain stably an oscillator by a 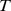-periodic (i.e. with a period of 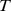 hours) sequence of Zeitgeber pulses, each pulse in the sequence must result in a delay or an advance that would compensate the period mismatch 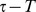. This requirement conditions the entrainment phase. Pulses must occur at the phase where the corresponding value of the PRC equals the mismatch 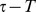. Thus, the largest mismatches that can be compensated correspond to the maximum and the minimum values of the PRC [1].

For the PRC of the strong oscillator in Figure 2 (left), we can compensate mismatches of up to 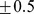 h. Negative mismatches of 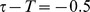 (clock faster than Zeitgeber) can be compensated by aligning the phases of the clock and the Zeitgeber in such a way, that the Zeitgeber pulse comes approximately at 270°. This allows for the desired phase delay of 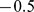 h, compensating for the faster clock. Analogously, for a clock slower than the Zeitgeber (mismatch 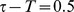 positive), the phases of the clock and the Zeitgeber must be aligned such that the light pulse arrives at approximately 90°, thus advancing the phase by about 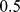 h. The same logic applies to the weak oscillator in Figure 2 (right). Here the PRC suggests that light pulses can compensate for larger mismatches up to approximately 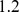 h.

We have now illustrated that the maximum and the minimum of the PRC correspond to the maximum and minimum mismatches 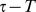, which can be compensated in entrainment, i.e. they correspond to the borders of entrainment range. The corresponding phases of pulses thus give the entrainment phases at the borders of the entrainment range. Both PRCs in Figure 2 are nearly sine-like, which implies that the maximum and minimum are nearly 180° apart and, consequently, the phase of entrainment can vary over a range of 180°. This is exactly the result from Figure 1 we were seeking to explain.

The 180° rule will be modified if the PRC is not well approximated by a sine-curve. Still, the maximum and minimum of the PRC determines the range of the entrainment phases. In “Methods” section, we provide mathematical details regarding the phase response curve/phase transition curve approach.

We emphasize that rodent and human PRCs resemble sine-curves for long durations of light pulses [22]. On the other hand, experimental PRCs based on short light pulses often clearly deviate from a sinusoidal shape [1]. Thus, a more general theory of phase response is required to deal with natural light-dark cycles, as described below.

#### Kuramoto phase equation

A serious limitation of the aforementioned PRC approach is the assumption that the Zeitgeber acts as a sequence of short pulses, each of them instantaneously introducing a phase delay or advance to the oscillator. For a continuously varying Zeitgeber, such as the daily variation of light intensity, this might be not the best representation. Fortunately, there is another theoretical tool which allows to overcome this limitation: The so-called Kuramoto equation describes the phase difference between the oscillator and its Zeitgeber for arbitrary Zeitgeber waveform [23]. The Kuramoto equation accounts for the Zeitgeber waveform by integrating its product with the PRC, compare Eq. (6). The integral produces sine-like waveforms even for PRCs deviating strongly from a sine-curve.

As outlined above and more detailed in “Methods” section, the phase of entrainment is essentially determined by the compensation of the mismatch 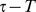 by the effective Zeitgeber strength. This implies that the Zeitgeber strength determines the allowed range of mismatches, for which entrainment occurs. This agrees with the intuition that larger Zeitgeber strength implies broader entrainment range (see next section).

The exact expression for entrainment phase 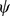 from the Kuramoto theory is
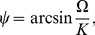
(2)where 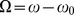 is the frequency mismatch between the Zeitgeber frequency 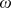 and endogenous frequency 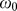. The parameter 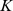 represents the effective Zeitgeber strength. For the phase of entrainment, Eq. (2) has the following consequences. First, it limits the choice of frequency mismatches 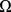 where entrainment is possible to the interval of 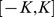 (recall that the 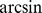-function is defined for arguments with the absolute value less than one). This results in the Arnold tongue, which is discussed in detail in the next section. Secondly, the unique values of the 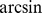 function span a range of 180°, which supports our 180° rule. The extrema of the entrainment phase are assumed at the border values of 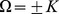, or, equivalently, in 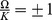. There, the 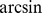 function produces values of ±90°, correspondingly.

Curiously, for a sinusoidal Zeitgeber, the expression for phase of entrainment obtained from the Kuramoto model coincides with the one for an oscillator with sinusoidal PRC under 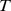-periodic sequence of light pulses. The explanation for this fact can be obtained by considering a continuous sinusoidal Zeitgeber as a superposition of many short pulses with a sinusoidal amplitude. A generalization of PRC theory leads to an integral of the product of PRC and the Zeitgeber, see section “Methods”, compare also [24]. When any of those two are sine-functions, the integral produces another sine-function, whose maximum and minimum are necessarily 180° apart. This again results in a 180° range of entrainment phase within the entrainment range. The Kuramoto theory can be thus also used to describe and predict the response of a circadian oscillator with a non-sine PRC to a periodic Zeitgeber.

#### Periodically driven damped oscillator

It has been shown that linear damped oscillators can be adequate models for the circadian rhythms in single cells [25]. This simple model allows an exact calculation of the phase difference between the external driving force and the oscillator, see “Methods” section.

When the period of the driving Zeitgeber is close to the intrinsic period of the oscillator, a resonance occurs – an increase of the amplitude of the driven oscillations. At the same time, the phase difference between Zeitgeber and the oscillator shows an interesting behavior. For a slow Zeitgeber (with 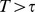), the phase difference is nearly zero – the oscillator and the Zeitgeber are in phase. In other words, the forced oscillator can follow the slow driver without any delay. On the other hand, if the Zeitgeber is fast compared to the oscillator (with 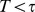), the phase difference 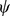 approaches 180° and the oscillator lags in antiphase behind the Zeitgeber. Again, between a fast and a slow Zeitgebers the phase difference can vary over a range of 180°, which nicely agrees with the aforementioned 180° rule.

For periodically driven damped oscillator with the eigenfrequency 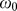 and damping rate 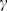, the phase difference 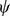 between driving force with frequency 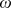 and oscillator is given by
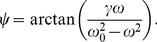
(3)


The 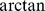 function produces values in the range of [0° ,180°]. The lower limit 0° is achieved when the argument of the 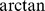 function becomes close to zero (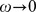), i.e. when Zeitgeber is much slower than the endogenous clock. If, on contrary, 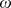 is much larger than 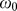, the argument of the 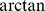 function becomes negative and 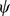 assumes the value of 180°. This is exactly our 180° rule – the phase difference varies over a range of 180°. Even though a driven linear oscillator has not an entrainment range in a strict sense, there is still a 180° phase difference between the oscillator response to slow and fast Zeitgeber periods.

In summary, three different approaches indicate that for strong oscillators, a phase range of about 180° can be expected within the entrainment range. This is not a strict rule since deviations from sinusoidal waveforms can cause variations of the 180° range.

### Entrainment for Variable Zeitgeber Strength

So far, we have considered Zeitgebers with a constant strength. A Zeitgeber can vary in its strength, like the amount of perceived daylight over different seasons or on different geographical latitudes [2]. The Zeitgeber strength, among other factors, determines the phase of entrainment. The main result of this section is that the 180° rule approximately holds for any Zeitgeber strength. As Zeitgeber strength increases, entrainment range spreads over larger mismatches 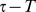 and so does the observed 180° flexibility of entrainment phase.

**Figure 3:**
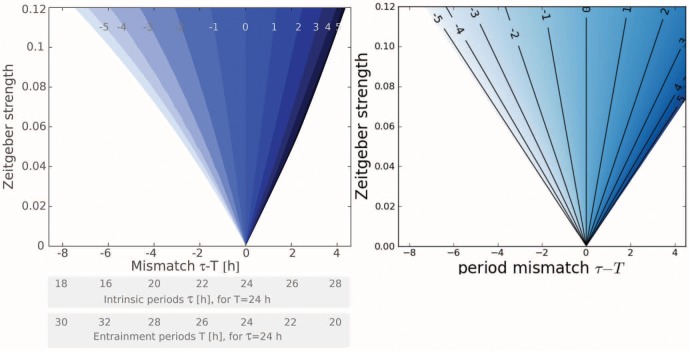
Phases of entrainment within the entrainment regions. Left: Numerical simulations of amplitude-phase model. Right: Entrainment phases from Eq. (2) derived from the Kuramoto phase equation. In both cases the entrainment phase varies from −6 h to 6 h between the borderlines of entrainment. Note that the lines with 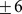 h are very close to those with 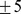 h and are not marked separately for the sake of clarity of the graphical representation.

Plotting the entrainment range versus increasing Zeitgeber strength produces a wedge-shaped entrainment region, see Figure 3, termed “Arnold tongue” (referring to the Russian mathematician V.I. Arnold). As intuitively expected, small Zeitgeber strength can overcome only small mismatches 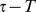. With increasing Zeitgeber strength, the Arnold tongue opens up and entrainment ranges over several hours can be observed [15], [26]. In Figure 3, we plot the phase of entrainment inside the Arnold tongue by different grades of blue. Close to the borders of the tongue, the entrainment phase assumes values of about 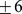 h. For small Zeitgeber strength, the phase of entrainment can vary a lot for small period mismatch changes due to a narrow entrainment range. This implies a high sensitivity of entrainment phase to mismatch 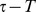. Contrarily, for large Zeitgeber strength, the entrainment range is large and the sensitivity of entrainment phase to mismatch is lower.

The effective Zeitgeber strength scales reciprocally with the oscillator amplitude [1], [10], [27]. Thus, the interaction of a given oscillator with a relatively small Zeitgeber strength is similar to the behavior of a strong oscillator. On the other hand, a strong Zeitgeber acting on a reference oscillator resembles a weak oscillator. Near the tip of the Arnold tongue where the entrainment range is narrow, the situation corresponds to entrainment of a strong oscillator. For large Zeitgeber strength, the entrainment range is wider, bearing a similarity to a weak oscillator.

Based on the Kuramoto equation, we show in “Methods” section that the phase of entrainment depends only on the ratio between normalized Zeitgeber strength 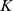 and mismatch 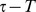. The condition for a constant entrainment phase can be expressed as
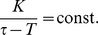



In coordinates “Zeitgeber strength – mismatch” 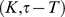, this is an equation for a straight line, going through the origin 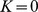, 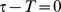. This finding nicely corresponds to the numerically obtained Figure 3, showing the lines of constant phases (isophases) as nearly straight lines, originating at zero Zeitgeber and mismatch. The above result implies that the phase of entrainment remains unchanged, if both Zeitgeber strength and mismatch are varied proportionally to each other.

### Entrainment Phase Flexibility and Chronotypes

We can use our previous theoretical results to explain how small variations in endogenous periods can result in large variations in chronotype. The endogenous period in human populations has been found to be fairly precise [28] with a standard deviation of about 12 minutes [29]. On the other hand, the mid-sleep time associated with entrainment phase 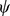 has been found to have a much larger variability of 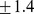 h [9]. It has been shown in [30] that the phase of entrainment 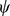 is strongly determined by the intrinsic period 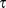. As an extreme example of high entrainment phase sensitivity, a patient with Familial Advance Sleep Phase Syndrome (FASPS) with a moderate mismatch 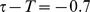 h has a 4 hour phase advance [19]. A question arises: How is it possible that a few minutes faster clock advances activity rhythms by more than an hour?

**Figure 4:**
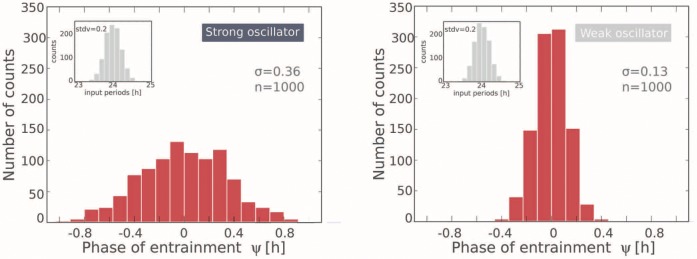
Flexibility of entrainment phases 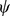 due to small variations of the endogenous period. The inserts show normally distributed periods with a standard deviation of 0.2 h. Simulations of amplitude-phase models illustrate the flexibility of entrainment phases for strong oscillators (left) compared to weak oscillators (right).

An answer is given by inspection of Figure 1 (a): for oscillators with a narrow entrainment range the slope of the function 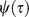 is quite large. Consequently, small differences in 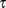 lead to large changes of the entrainment phase. For an entrainment range of 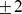 h we obtain, for instance, a slope of 3 due to the 12 h range of the entrainment phase. As an approximation, we can use the relation
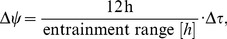
(4)connecting period changes 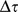 to variations of the entrainment phase 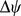. The numerator is fixed to be about 12 h according to the 180° rule. Consequently, the denominator governs the slope. A narrow entrainment range leads to large slopes of the function 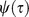. Figure 4 from the Aschoff survey [8] summarizes the experimentally found relations between the phase of entrainment and the range of entrainment in 15 species. From that survey slopes between 0.5 (for unicellular organisms) and 4.5 (for birds) can be extracted.

We illustrate our explanation of the relation between 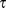 and chronotype in Figure 4. We simulated two ensembles with strong and weak oscillators, correspondingly, with a Gaussian distribution of 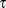 with the mean of 24 h and a standard deviation of 0.2 h. For strong oscillators (Figure 4, left) we obtain a wide range of entrainment phases whereas a weak oscillator (Figure 4, right) leads to a narrow distribution of 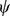. The standard deviation of entrainment phase of strong oscillators was found to be nearly three times larger than for weak ones. Thus, for strong oscillators including the human clock, small variations in 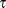 lead to highly variable entrainment phases.

## Discussion

### Flexibility of Entrainment Phase

We provide a conceptual framework how oscillator properties control the entrainment phase. Strong oscillators with a narrow entrainment range exhibit more flexible entrainment phases (see Figure 4). This implies that small variations of the endogenous period 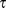 lead to different phases, associated with different chronotypes.

The core of our theory is the “ 180° rule” formulated already by Wever [7]: under general assumptions, the entrainment phase varies within the entrainment range by 180° (or 12 h given a period of 24 h). This rule is rigorously derived using 3 different approaches: (i) phase response curves, (ii) phase model (Kuramoto equation) and (iii) resonance theory. Numerical simulations of limit cycle models as in Figures 1 and 3 confirm the 180° rule. As discussed in “Methods”, Eq. (6), the integral of the product of Zeitgeber and PRC determines the phase dynamics. Even for non-sinusoidal PRCs, the 180° rule can hold, as long as the integral in Eq. (6) produces a sine-like function. Indeed, already Aschoff and Pohl [8] suggested that the 180° rule is approximately valid in many organisms, even though most PRCs deviate from a sinusoidal shape [1].

### Generalizations of the Theory

Our central equation (4) that connects phase variations 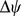 to period changes 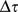 and the entrainment range is a linear approximation of the 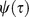 functions. Inspection of Figure 1 reveals that there are clear deviations from linearity. Sigmoidal functions 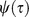 have been reported earlier [8]. Our calculations predict curved 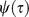 relationships near the borderlines of the entrainment range. The theory of driven damped oscillators leads directly to sigmoidal functions. Sinusoidal PRCs and Kuramoto phase equation result in 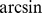 functions resembling the simulation results in the left graph of Figure 1. Consequently, our theory is also capable to explain deviations from a linear function 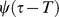, which have been experimentally found [7], [8], [31].

In Figure 3, we have implicitly assumed that the intrinsic period 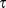 is not changing with Zeitgeber strength. It is known, however, that light intensity may change the endogenous period 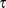 [6], [31]. This would lead to skewed Arnold tongues. Furthermore, seasonal variations implicitly change Zeitgeber strength and induce period and entrainment phase variations [31], [32]. These issues will be addressed systematically in a forthcoming study.

### Theoretical Predictions

As visualized in Figure 3, our theory predicts the dependence of the entrainment phase on mismatch 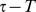 and Zeitgeber strength. For 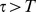, an increase of the Zeitgeber strength leads to earlier entrainment phases. This effect has been shown recently in temperature entrainment studies [10]. The phase of entrainment of lung tissue was decreased by 3 h by increasing Zeitgeber strength (compare Figure S5 in [10]). Comparable predictions relate light intensity and chronotypes [33]. For night owls with 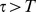 we expect that more light leads to earlier phases. Considering the left half of Figure 3, large Zeitgeber strength leads to later phases for 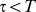. Altogether, the theory predicts that stronger Zeitgebers lead to narrower distributions of chronotypes. This prediction can be tested comparing chronotype distributions from different countries or in summer and winter [34].

### Evolutionary Aspects of Entrainment Phase Flexibility

Temporal coordination of physiology and behavior with extrinsic Zeitgebers leads to evolutionary benefits of a properly functioning circadian clock. In particular, a suitable phase of entrainment most likely provides selective advantages. It is by no means obvious whether or not a stable entrainment phase as observed for weak oscillators or a flexible phase for strong oscillators is advantageous. For example, bird and butterfly navigation can be supported by a phase marker that is precisely aligned to Zeitgeber phase [35], [36]. If an organism can track noon independent of season and latitude, the highest point reached by the sun always defines south direction in the northern hemisphere.

In many other situations a flexible phase of entrainment is required. The prime example is the adaptation to seasonal changes. Frequently the circadian clock allows to track dusk and dawn. This can be achieved partly by masking [37], but the observation of activity peaks tracking sunrise and sunset indicates successful adaptations of the entrainment phase to seasonal changes [1], [2].

Across many organisms narrow and wide ranges of entrainment are observed [8]. This implies that the slopes of the function 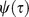 varies from 0.5 (unicellular organisms) up to 4 (vertebrates) since the “180° rule” associates entrainment ranges with the phase variability described by the slope 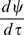. A wide entrainment range corresponds to a high responsiveness to Zeitgeber clues. For example, the clock of unicellular organisms is heavily influenced by inputs.

In mammals, a hierarchy of circadian oscillators exists. The suprachiasmatic nucleus (SCN) receives direct light input and orchestrates peripheral clocks [38]. The SCN is a quite strong oscillator with a relatively narrow range of entrainment. This has been shown recently for temperature as a universal Zeitgeber [10], [18] but applies also to light inputs since phase response curves have typically relatively small advance and delay portions [39]. In contrast, peripheral clocks have large PRCs and wide entrainment ranges [10]. These observations might reflect a design principle of mammalian clocks: the pacemaker subject to noisy inputs is quite strong whereas peripheral clocks constitute weak oscillators that can be easily entrained by neuronal and humoral signals or body temperature. The theory of entrainment phase control, as discussed in this paper, emphasizes that the robustness of the SCN implies flexible entrainment phases due to large slopes of the function 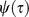. The wide spread of chronotypes reflects this large phase flexibility. Moreover, the sensitivity of the entrainment phase allows adaptation to seasons and latitudes. In a forthcoming study we will show how oscillator properties rule seasonal adaptations as observed in the classical work of Daan and Aschoff [2].

## Methods

Figures 1–4 are based on simulations of the amplitude-phase oscillator Eq. (1) with 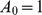. Periodic forcing is simulated by adding the term 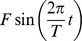 to the 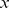-coordinate of the oscillator with Eq. (1) re-formulated in the Cartesian coordinates. The parameter 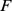 is termed “Zeitgeber strength” in Figure 3. The PRC in Figure 2 is obtained with pulses of strength 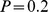 lasting 30 min. The entrainment phases visualized in Figure 3 are based on simulations with varying period mismatch and Zeitgeber strength. For each parameter set, 24 initial conditions were chosen equally distributed along the limit cycle. The median entrainment phase after 50 days was plotted using contour lines. Numerical simulations were performed in MATLAB (2007a, The MathWorks, MA, Natick).

Below we consider generic models of periodically driven oscillators. We present calculations showing that under quite general assumptions, the entrainment phase varies over about 180° (or 12 h for circadian periods). The derivation of this “180° rule” points also to limitations and generalizations of this finding.

### Sinusoidal Phase Response Curve

In many cases the effects of external driving forces can be studied successfully using Phase Response Curves (PRCs) [20], [31], [40]. If the relaxation of a perturbation is fast compared to the period of the oscillator, PRCs can be used to calculate the phase of entrainment for periodical stimulations [41]. This discrete approach to the entrainment of circadian clocks is mathematically related to stable fixpoints of the associated Phase Transition Curve (PTC).

For a sinusoidal PRC we find the following PTC [15]:
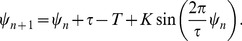
(5)Here the phase variable 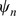 is normalized to the range 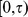, where 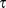 is the endogenous period. Further, 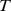 denotes the period of the Zeitgeber and the parameter 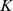 represents the effective Zeitgeber strength.

**Figure 5:**
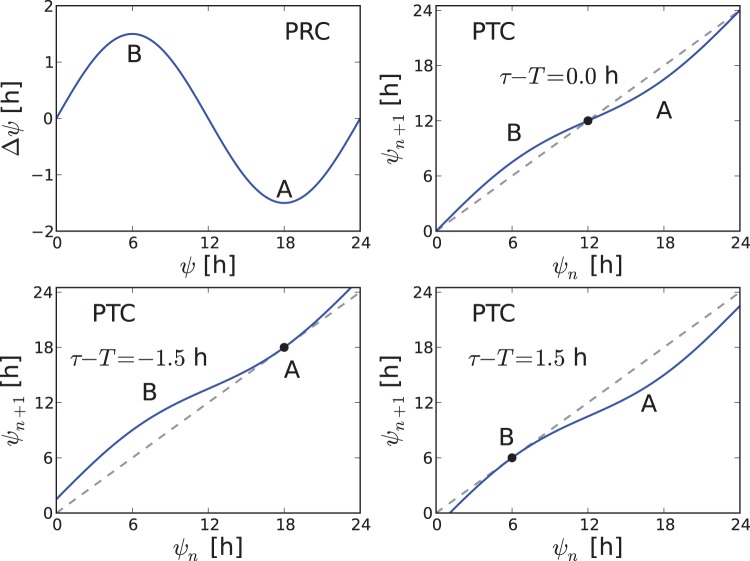
Sinusoidal phase response curve (PRC) and associated phase transition curves (PTCs) according to Eq. (5). Applying 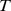-periodic pulses, stationary entrainment phases are given by the intersections of the PTC with the diagonal 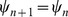 [15]. Upper graphs: Vanishing frequency mismatch 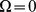 leads to a stable entrainment phase 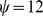 h. Lower graphs: Period mismatches 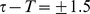 correspond to the borderlines of the entrainment range. The corresponding entrainment phases of 18 h and 6 h are associated to the extrema of the sine-function and are 12 h (or 180° ) apart.

The upper graphs in Figure 5 show a sinusoidal PRC and its associated PTC for the frequency detuning 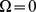 and Zeitgeber strength 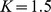. The PTC can be considered as an iterated map [15], i.e. subsequent perturbations lead to a series of iterated phases 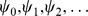. Fixpoints of this map are given by the intersection with the diagonal 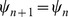. A stable fixpoint (e.g. the full circle at 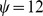 h in Figure 5) corresponds to the entrainment phase. Increasing the frequency mismatch 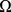 leads to a loss of entrainment, since there are no more intersections of the PTC with the diagonal. This happens at the marked point 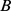 via a saddle-node bifurcation (see [15] for details). The disappearance of a stable fixpoint marks the borderline of the entrainment region (compare Figure 1). Similarly, negative values of the frequency mismatch 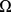 lead to a loss of entrainment at point 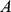. The critical entrainment phases at 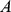 and 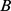 correspond to the extrema of the PRCs in Figure 2, since the PTC is essentially a rotated version of the PRC [41]. For sinusoidal PRCs, these extrema are half a period (or, equivalently, 180°) apart. This implies that the phase entrainment varies by 12 h as illustrated in Figure 1.

Note that Figures 1–4 are obtained from simulations of amplitude-phase oscillators with sinusoidal periodic forcing. Consequently, the PTC-theory discussed above is only approximately valid. Thus, the entrainment range is larger and more asymmetric than the advances and delays in Figure 2 in the main text. A more general approach of periodically driven limit cycle has been developed by [23] and will be discussed below.

### Entrainment Phase of Periodically Driven Limit Cycles

Kuramoto [23] studied extensively the phase dynamics of driven oscillators. His theory is intimately related to the continuous approach of Aschoff [6]. The following differential equation for the phase difference 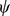 between the periodic force and oscillator can be derived:
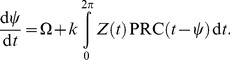
(6)Here, 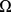 has the same meaning as the frequency mismatch between the Zeitgeber and the oscillator as above. The integral represents the cumulative action of the Zeitgeber 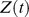 as “sensed” by the PRC. If PRC or Zeitgeber are sinusoidal, the integral can be computed analytically and the above equation simplifies to what we know nowadays as the Kuramoto equation:



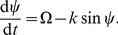
(7)The equation looks quite similar to the PTC discussed in the previous section. Again the extrema of the sine-function (180° apart) correspond to the borderline of the entrainment range. A stationary phase determined by the condition 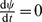 equals
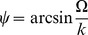
within the entrainment range 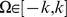. This implies that the entrainment phase varies again by 180° within the entrainment range. The 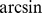-function resembles the simulation result in Figure 1, left graph. In the middle, its slope 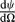 is approximately given by 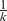 , i.e. for small entrainment ranges large slopes occur.

Due to the periodicity of Zeitgeber and PRC, the integral in Eq. (6) is a 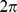-periodic function in phase 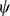. A special case of certain shapes of Zeitgeber and PRC is worth to be discussed separately: If one of the functions 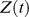 or PRC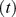 contains no higher harmonics (i.e. is either a pure sine or a cosine function or a linear superposition of such), the integral would contain no higher harmonics either. As a consequence, the 180° rule would hold exactly in this case. Under a milder assumption that both Zeitgeber and PRC do contain higher harmonics, the integration would suppress them.

In comparison to the PRC approach, the Kuramoto theory provides a more general framework for entrainment. The entrainment phase can be determined for different Zeitgebers and PRCs. According to the above calculation, any combination of Zeitgeber and PRC that produces a phase equation similar to Eq. (7) implies the 180° rule. In summary, the phase equation according to Kuramoto [23] leads to a similar conclusion as the PRC approach. Narrow entrainment ranges imply strong dependences of the entrainment phase 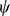 on the mismatch 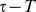.

### Periodically Driven Damped Oscillator

**Figure 6:**
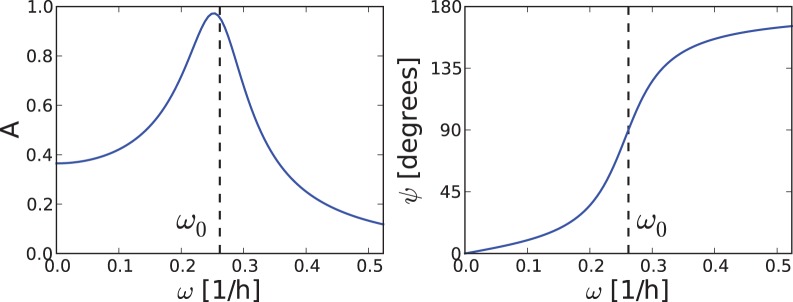
Amplitude and phase of a periodically driven weakly damped oscillator. The amplitude (left) shows a resonance peak near the intrinsic frequency 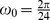. Near the resonance the phase difference between the oscillator and the driver is changing by about 180° (right).

Even though the circadian clock has been proven to be a self-sustained oscillator, weakly damped oscillations exhibit somewhat similar features when periodically driven [42]. Amplitudes increase via resonance and phases vary with the mismatch between intrinsic angular frequency 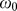 and driver frequency 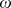. For a linear oscillator
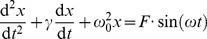
(8)the amplitude 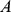 and the phase 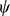 of the driven oscillation can be calculated



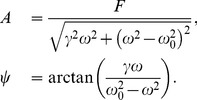
(9)
Figure 6 shows the corresponding functions. For slow external frequencies 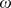, the forced oscillator is in phase with the driving force, whereas it lags behind it by 180° for fast drivers (with high 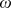). This model shows large phase shifts near the resonance resembling entrainment phase variations of driven limit cycle oscillators. It has been shown recently that weakly damped oscillators are fairly good approximations of circadian single cell rhythms [25]. If the amplitude shows resonance behavior as observed in lung tissues [10] and skin cells [43], the theory of weakly damped oscillators can serve as a reasonable approximation.

We do not claim that the “180° rule” derived here can be considered as a universal law. Obviously, deviations from a sinusoidal PRC shape, phase resetting by large pulses, and periodically driven relaxation oscillators require a more general theory. Nevertheless, the “180° rule” is supported by quite different mathematical approaches (PTC, Kuramoto’s phase equation, resonance theory) and can be successfully applied to strong oscillators with type-1 PRCs and narrow entrainment ranges. These assumptions are reasonable for many vertebrates [8] including humans [21].
